# Accelerated Current Decay Kinetics of a Rare Human Acid-Sensing ion Channel 1a Variant That Is Used in Many Studies as Wild Type

**DOI:** 10.3389/fnmol.2019.00133

**Published:** 2019-05-24

**Authors:** Anand Vaithia, Sabrina Vullo, Zhong Peng, Omar Alijevic, Stephan Kellenberger

**Affiliations:** Department of Pharmacology and Toxicology, University of Lausanne, Lausanne, Switzerland

**Keywords:** ASIC, variant, mutation, kinetics, patch-clamp, voltage-clamp fluorometry

## Abstract

Acid-sensing ion channels (ASICs) are neuronal Na^+^-permeable ion channels that are activated by extracellular acidification and are involved in fear sensing, learning, neurodegeneration after ischemia, and in pain sensation. We have recently found that the human ASIC1a (hASIC1a) wild type (WT) clone which has been used by many laboratories in recombinant expression studies contains a point mutation that occurs with a very low frequency in humans. Here, we compared the function and expression of ASIC1a WT and of this rare variant, in which the highly conserved residue Gly212 is substituted by Asp. Residue 212 is located at a subunit interface that undergoes changes during channel activity. We show that the modulation of channel function by commonly used ASIC inhibitors and modulators, and the pH dependence, are the same or only slightly different between hASIC1a-G212 and -D212. hASIC1a-G212 has however a higher current amplitude per surface-expressed channel and considerably slower current decay kinetics than hASIC1a-D212, and its current decay kinetics display a higher dependency on the type of anion present in the extracellular solution. We demonstrate for a number of channel mutants previously characterized in the hASIC1a-D212 background that they have very similar effects in the hASIC1a-G212 background. Taken together, we show that the variant hASIC1a-D212 that has been used as WT in many studies is, in fact, a mutant and that the properties of hASIC1a-D212 and hASIC1a-G212 are sufficiently close that the conclusions made in previous pharmacology and structure-function studies remain valid.

## Introduction

**Figure 1:**
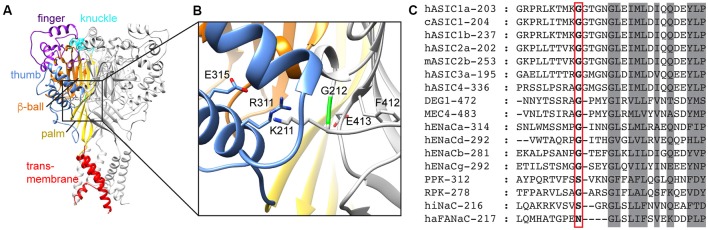
Domain organization of acid-sensing ion channel (ASIC) and conservation of G212. **(A)** Structural representation of an ASIC trimer, highlighting the different domains in one of the three subunits by different colors, for the finger (purple), knuckle (cyan), thumb (blue), β-ball (orange), palm (yellow) and transmembrane parts (red). The model is based on the open chicken ASIC1a (cASIC1a) structure (Baconguis et al., 2014). **(B)** Details of the area around G212. Color code as in **(A)**. **(C)** Sequence alignment of representative Epithelial Na^+^ channel (ENaC)/degenerin family members. The number of the first displayed residue is indicated after the name, and G212 is highlighted with a red frame.

A rare variant of the human acid-sensing ion channel 1a (hASIC1a) has been used as “wild type” (WT) in many functional studies involving recombinant expression of ASICs, including those carried out by our laboratory. Here, we investigate the differences between this variant, hASIC1a-D212, and the hASIC1a WT that has a Gly residue at position 212. ASICs are neuronal, Na^+^-conducting ion channels expressed in the central and peripheral nervous system (Wemmie et al., 2013; Yang and Palmer, 2014; Kellenberger and Schild, 2015). Their activation by extracellular acidification leads to neuronal depolarization (Deval et al., 2003; Vukicevic and Kellenberger, 2004). A sustained acidification leads to a transient ASIC current since these channels enter a non-conducting desensitized state rapidly after opening. ASICs contribute to fear sensation, neurodegeneration after ischemic stroke, to learning and to pain sensation (Wemmie et al., 2013; Kellenberger and Schild, 2015). Of the six different ASIC subunits, ASIC1a, ASIC1b, ASIC2a, ASIC2b and ASIC3 can form homo-or heterotrimeric channels (Jasti et al., 2007; Wemmie et al., 2013; Bartoi et al., 2014). The subunit composition determines the biophysical properties of the channel, such as pH dependence, current kinetics and presence or absence of a sustained current fraction (Wemmie et al., 2013; Grunder and Pusch, 2015; Kellenberger and Schild, 2015). Each ASIC subunit contains intracellular N- and C-termini, two transmembrane α helices, and a large extracellular loop. Crystal structures of chicken ASIC1a (cASIC1a), whose sequence shares ~90% homology with hASIC1a, were obtained in conformations corresponding to the desensitized, the open and the closed state (Jasti et al., 2007; Gonzales et al., 2009; Baconguis and Gouaux, 2012; Dawson et al., 2012; Baconguis et al., 2014; Yoder et al., 2018). They show that the shape of a subunit is comparable to a hand, with the domains palm (yellow in Figure 1A), β-ball (orange), knuckle (cyan), finger (purple) and thumb (blue); the transmembrane domain would correspond to the forearm (Jasti et al., 2007). ASICs are the target of rather nonspecific small molecule inhibitors such as diminazene (Chen et al., 2010) and amiloride (Waldmann et al., 1997), of modulators such as 2-guanidine-4-methylquinazoline (GMQ; Yu et al., 2010) and of several high affinity toxins (Baron and Lingueglia, 2015; Rash, 2017).

We have recently realized that a hASIC1a clone, which is used by many laboratories in studies employing recombinant expression, contains a substitution by Asp of the conserved residue Gly212. A large proportion of published articles using hASIC1a was done with this clone (Supplementary Table S1). This clone [GenBank accession number U78181 (García-Añoveros et al., 1997)] is, however, a rare variant; in >99% of humans, the residue at position 212 is a Gly. It is not clear whether the presence of Asp at position 212 is due to a cloning artifact, or whether the individual from which the clone originates carried this mutation. According to the database of the Exome Aggregation Consortium^1^ (Lek et al., 2016) the frequency of Asp instead of Gly at position 212 is 8.26 × 10^−6^. In the proximity of Gly212, there are two other Gly residues in ASIC1a, at positions 213 and 217 (Figure 1C). The Gly pair at positions 212 and 213 is highly conserved in ASICs (Supplementary Figure S1), and one of the two Gly residues is present in all mammalian Epithelial Na^+^ channel (ENaC)/degenerin (DEG) family members. Since this region is otherwise not so well conserved among the superfamily, it is not possible to say whether Gly212 or Gly213 is conserved in the other mammalian ENaC/DEG subfamilies. Gly217, in contrast, is conserved in all ENaC/DEG family members. In the structural model of ASIC1a, Gly212 is located on the β-ball domain at a subunit interface, facing the thumb domain of a neighboring subunit, close to a proposed chloride binding site (Jasti et al., 2007; Figure 1B). In the present study, we compared the function and pharmacology of the WT hASIC1a-G212 and the mutant hASIC1a-D212 and found highly similar properties regarding many aspects. hASIC1a-G212 displayed, however, a higher current amplitude per surface-expressed channel, and slower desensitization kinetics.

## Materials and Methods

### Molecular Biology

For the expression in *Xenopus* oocytes, the hASIC1a sequence (García-Añoveros et al., 1997) was sub-cloned into a pSP65-derived vector containing 5′ and 3′ non-translated sequences of β globin to improve the stability and the expression in *Xenopus laevis* oocytes. For the expression in chinese hamster ovary (CHO) cells, the human and mouse ASIC1a sequences were sub-cloned into the peak8 vector (Edge Biosystems, Gaithersburg, MD, USA). Amino acid substitutions were generated by site-directed mutagenesis using KAPA HiFi HotStart PCR polymerase (KAPA Biosystems), using the Quikchange approach. Mutations were verified by sequencing (Synergen Biotech). *In vitro* transcription was performed using the mMESSAGE mMACHINE SP6 kit (Ambion/Life Technologies).

### Mammalian Cell Culture and Transfection

For the experiments with hASIC1a-D212 and mouse ASIC1a WT, CHO cells stably expressing these constructs were used (Poirot et al., 2004). For expression of hASIC1a-G212 and mASIC1a-G212D, CHO cells were transiently co-transfected with complementary deoxyribonucleic acid (cDNA) of EGFP together with the ASIC construct, by using Rotifect (CarlRoth). For expression of heteromeric ASICs, cDNA of hASIC1a-D212 and hASIC1a-G212 was transiently co-transfected with hASIC2a, EGFP and salmon sperm DNA into CHO cells. The ASIC1a/ASIC2a cDNA ratio for the transfections was 1:1. CHO cells were cultured in DMEM/Nutrient Mixture F-12 with GlutaMAX^TM^ medium supplemented with 10% fetal bovine serum (FBS, ThermoFischer Scientific) and 1% Penicillin-Streptomycin (5,000 U/mL, ThermoFischer Scientific) and the cells were grown at 37°C under 5% CO_2_ atmosphere. Stable cell lines were supplemented with puromycin (10 μg/ml) to maintain the stable expression of ASICs.

### Oocyte Handling and Injection

All experiments with *Xenopus laevis* oocytes were carried out in accordance with the Swiss federal law on animal welfare and had been approved by the committee on animal experimentation of the Canton de Vaud. After surgical removal, healthy stage V and VI oocytes of female *Xenopus* frogs were treated with collagenase for isolation and defolliculation. They were subsequently injected with 50 nl (0.02–0.8 μg/μl) of cRNA. After injection, they were kept at 19°C in Modified Barth’s Solution (MBS) composed of (mM): 85 NaCl, 1 KCl, 2.4 NaHCO_3_, 0.33 Ca(NO_3_)_2_, 0.82 MgSO_4_, 0.41 CaCl_2_, 10 HEPES and 4.08 NaOH. Experiments were performed 24 h to 48 h after injection.

### Electrophysiological Measurements

#### Whole-Cell Patch-Clamp of Mammalian Cells

Whole-cell patch-clamp recordings were carried out in stable cell lines or after 48 h of transient transfection at −60 mV with an EPC-9 amplifier (HEKA Electronics). The solution exchange was carried out using the MPRE8 perfusion head and electrovalves (Cell MicroControls). The sampling interval was set at 1 ms and the current filtering was set to 3 kHz. Patch pipettes (3–4 MΩ) were pulled from borosilicate glass with filament (WPI Precision Instruments, UK) using the vertical dual-stage pipette puller PC10 (Narishige). Compensation of the series resistance was set to 70%–90%. The standard extracellular solution contained (in mM) 140 NaCl, 4 KCl, 2 CaCl_2_, 1 MgCl_2_, 10 MES, 10 HEPES, 10 Glucose, and pH was adjusted to 7.4 with NaOH. The intracellular solution contained (in mM) 90 K-Gluconate, 10 NaCl, 10 KCl, 60 HEPES, 10 EGTA, and the pH was adjusted to 7.3 with KOH. In the experiments with 100 nM extracellular Ca^2+^ concentration, 10–20 mM of a Ca^2+^ chelator (EGTA at pH >7.2, EDTA at pH ≤7.2) was included and the total Ca^2+^ concentration was adjusted to obtain a free Ca^2+^ concentration of 100 nM according to Maxchelator (Bers et al., 2010). In the experiments with anion substitution, NaCl was replaced by NaSCN, and K^+^ was included as KOH, Ca^2+^ as Ca-Gluconate, and Mg^2+^ as MgSO_4_. In these solutions, the pH was adjusted to pH 6.0 with 10% acetic acid and to pH7.4 with NaOH.

#### Outside-Out Patches

For outside-out patches, the same solutions as for whole-cell measurements were used. Coverslips containing transfected CHO cells were maintained during recording with external solution of pH7.4. Outside-out patches were excised with 4–6 MΩ borosilicate glass pipettes from transfected cells. The proton-evoked currents were recorded at −60 mV, at a sampling rate of 50 μs and low-pass-filtered at 2.9 kHz. Rapid pH changes (every 8 s) were carried out using a Piezo-controlled fast application system with a double-barrel application pipette that enables solution exchange (MXPZT-300L; Siskiyou, Grants Pass, OR, USA).

#### Measurements From *Xenopus* Oocytes

Standard recording solutions contained (in mM) 110 NaCl, 2 CaCl_2_, and 10 HEPES for pH ≥ 6.8. For solutions with a pH < 6.8, HEPES was replaced by 10 mM MES. The pH was adjusted using NaOH or HCl. Whole-cell currents from *Xenopus* oocytes were recorded by two-electrode voltage clamp (TEV-200A; Dagan Corporation) at −60 mV or as indicated, using Chartmaster software (HEKA Electronics) at a sampling rate of 1 ms and low-pass filtering at 2 kHz. Oocytes were placed in a RC-26Z recording chamber (Warner Instruments) and impaled with two glass electrodes filled with 1 M KCl, with a resistance of <0.5 MΩ. Oocytes were perfused at a rate of 5–15 mL/min. All experiments were performed at room temperature (20–25°C). To determine the pH dependence of the channel activation and steady-state desensitization (SSD), oocytes were exposed to a conditioning pH solution (generally pH7.4 in activation protocols) and stimulated with an acidic pH for 5–10 s, or as indicated, once per minute.

#### Voltage-Clamp Fluorometry

All voltage-clamp fluorometry (VCF) experiments were carried out on fluorophore-labeled cysteine mutants. Oocytes were labeled in the dark with 5 μM CF488- (Biotium) or AlexaFluor488 C-5 maleimide (Invitrogen) for 15 min at room temperature. VCF experiments were done in a RC-26Z recording chamber (Warner Instruments). The VCF setup was equipped with an Intensilight mercury lamp (C-HGFI; Nikon). A 40× Nikon oil-immersion objective (CFI Plan Fluor; Nikon) was used to detect the fluorescence signal emitted by the labeled oocytes. The optical signal was measured by a photodiode (S1336-18BQ; Hamamatsu Photonics) coupled to the headstage of an amplifier (List EPC-7; HEKA). An offset device was used to adjust and measure the offset of the signal, allowing the measurement of the total fluorescence intensity. Changes in fluorescence intensity (ΔF) were normalized to the total fluorescence signal (F).

### Electrophysiology Data Analysis and Statistics

Data were analyzed with the software FitMaster (HEKA Electronics) and with Origin PRO (OriginLab Corp., Northampton, MA, USA). pH response curves for H^+^ activation were fitted with a Hill function: I = I_max_/(1 + (10^−pH50^/10^−pH^)^nH^), where I_max_ is the maximal current amplitude, pH_50_ is the pH inducing 50% of the maximal current amplitude, and nH is the Hill coefficient. SSD curves were fitted with an analogous equation. Time constants of desensitization were determined by fitting the decay time of current traces to a mono-exponential function. The results are presented as mean ± SEM. They represent the mean of *n* independent experiments on different cells. Statistical analysis was performed with *t*-test where two conditions were compared, or with One-way ANOVA followed by Dunnett’s or Tukey multiple comparisons test, or as indicated (Graphpad Prism 6).

### Surface Protein Biotinylation and Western Blot

**Figure 2:**
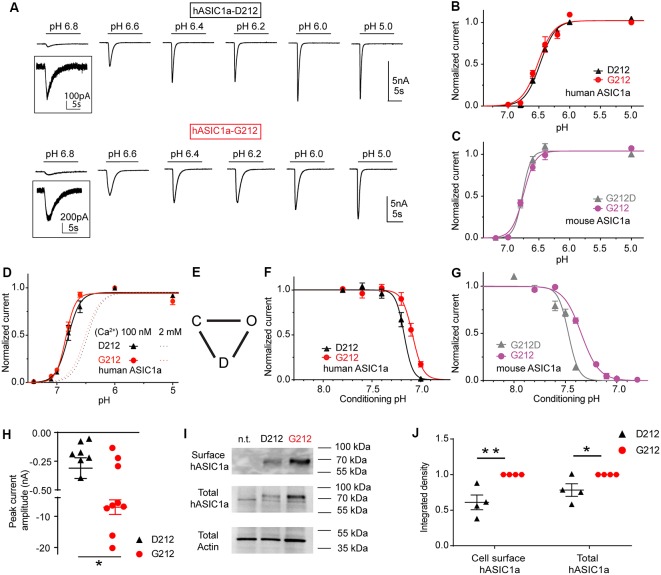
The G212D substitution changes the pH dependence of steady-state desensitization (SSD). These experiments were obtained with whole-cell patch-clamp of chinese hamster ovary (CHO) cells expressing the indicated channels at a membrane potential of −60 mV. **(A)** Current traces of human ASIC1a (hASIC1a)-D212 (top) and hASIC1a-G212 activation (bottom), obtained with stimulation pH values as indicated, from a conditioning pH of 7.4. The acidic pH was applied for 10 s every 40 s. **(B)** Normalized current response as a function of the stimulation pH of hASIC1a-D212 and -G212, *n* = 11–12. **(C)** Normalized current response as a function of the stimulation pH of mouse ASIC1a-G212 and -G212D, *n* = 5–7. **(D)** Normalized current response as a function of the stimulation pH of hASIC1a-D212 and -G212, obtained with a low Ca^2+^ concentration in the extracellular stimulation solution (100 nM free Ca^2+^), as opposed to 2 mM in all other experiments, *n* = 5–6. **(E)** Kinetic scheme of ASIC function, indicating the closed, open and desensitized state. **(F)** SSD curves of hASIC1a-D212 and -G212, obtained by application of the indicated conditioning pH for 1 min, followed by activation at pH 5 for 5 s. Sweeps with conditioning pH of 7.4 were alternated with sweeps with test conditioning pH. The normalized current amplitude is plotted as a function of the conditioning pH, *n* = 5. **(G)** SSD curves of mASIC1a-G212 and -G212D, obtained and presented as described above, *n* = 5–7. **(H)** pH5.0-induced peak current amplitude after transfection of 60 ng hASIC1a DNA/35 mm dish, *n* = 9. **(I)** Representative Western blot images of ASIC1a cell surface biotinylated protein (top), total hASIC1a protein (middle) and the actin from total lysate (bottom); n.t., non transfected. Actin was used as the control for sample preparation and loading. **(J)** Normalized integrated density of the bands of the total and cell surface-expressed ASIC1a (*n* = 4) *p < 0.05; **p < 0.01.

These experiments were carried out as described elsewhere (Jiang et al., 2010). Briefly, cells were washed 48 h after transfection with PBS-CM (in mM, 137 NaCl, 2.7 KCl, 8 Na_2_HPO_4_, 2 KH_2_PO_4_, 0.1 CaCl_2_, 1 MgCl_2_) at pH7.4 and then washed twice with PBS-CM at pH8.0. All procedures were performed on ice. Cells were labeled with 1 mg/ml of EZ-Link^TM^ Sulfo-NHS-SS-Biotin (Thermo Scientific) supplemented in PBS-CM at pH8.0 for 30 min. The biotinylation reaction was quenched by adding 10 ml of PBS-CM at pH7.4 containing 100 mM glycine for 10 min, followed by two washes with PBS-CM at pH7.4. Cell lysates were prepared by incubation for 15 min in cell lysis buffer containing 100 mM NaCl, 5 mM EDTA, 1% Triton X-100, 20 mM HEPES at pH7.4, supplemented with protease inhibitor cocktail. Cell lysates were clarified by centrifugation at 4°C for 20 min at 11,000 *g*. The total ASIC protein expression was determined by mixing 20 μl of the supernatant with 5 μl of 5× sample loading buffer [1.5 M Sucrose, 10% SDS, 12.5mM EDTA, 312 mM Tris pH8.8, 0.25% (w/v) bromophenol blue, 125 mM DTT], followed by heating at 95°C for 10 min. These samples were then used for SDS-PAGE and western blot analysis. The remaining supernatant was mixed with 50 μl neutravidin-agarose beads (Thermo Scientific) and incubated overnight at 4°C on a rotating wheel. Beads and supernatant were separated by centrifugation at 1,000 *g* for 3 min and washed five times with PBS-CM at pH7.4 containing 1% Triton X-100. The supernatant was discarded and 50 μl of 2× sample loading buffer [20% glycerol, 6% SDS, 250 mM Tris-HCl at pH6.7, 0.1% (w/v) bromophenol blue, 50 mM DTT] was added to each sample, followed by heating at 95°C for 10 min. Protein samples of 20 μl were separated on a 10% SDS-PAGE (running buffer: 27.5 mM Tris-base, 213 mM Glycine and 1% SDS) at 100V for 1.5 h. Samples were transferred to Protran^TM^ 0.2 μM nitrocellulose membranes (Amersham Biosciences) at 4°C, 100V for 2.5 h. After the transfer, the membrane was blocked by TBST (in mM, 137 NaCl, 2.7 KCl, 19 Tris base, 0.1% Tween 20) containing 5% BSA for 1 h. The membranes were exposed overnight at 4°C to anti-ASIC1 antibody (1:1,000, Alomone, Israel) in 1% BSA containing TBST buffer, washed three times, and were then exposed to Goat Anti-Rabbit IgG H&L horseradish peroxidase-linked secondary antibody (1:4,000, Abcam, Switzerland). To detect actin, the blots were exposed overnight at 4°C to Anti-Actin (1:1,000, Sigma Aldrich) in TBST buffer containing 1% BSA, washed three times and detected with Donkey anti-rabbit IgG horseradish peroxidase-linked secondary antibody (1:4,000, GE healthcare). Blots were exposed to secondary antibodies for 1 h at room temperature and washed three times. The signals were detected using the Fusion SOLO chemiluminescence system (Vilber Lourmat, Marne-la-Vallée, France) using SuperSignal^TM^ West Femto Maximum Sensitivity Substrate (Thermo Scientific). Actin was detected from the cell lysate (Figure 2I), but not from the surface fraction (not shown). The band intensities were quantified by the linear analysis method of the software, with the area of measurement kept the same for all samples of the same blot. Background noise was subtracted prior to determining the intensity occupied by individual bands.

### Reagents

GMQ was purchased from Sigma, and a fresh GMQ stock solution in standard extracellular solution was prepared every day; dilutions were then made from the stock solution. Psalmotoxin1 was purchased from Smartox Biotechnology (France); solutions were made daily from a 214 μM stock solution in water. Mambalgin1 was purchased from Peptides International (USA). Mambalgin solutions were made fresh daily from a 30 μM stock solution in water. 0.05% BSA (final concentration) was included in solutions containing the toxins. Amiloride hydrochloride (Sigma Aldrich) was dissolved in the normal extracellular solution at pH6.0 at 1 mM and kept at 4°C. Solutions at different amiloride concentrations were freshly made on the days of the experiments.

## Results

### Slower Current Decay Kinetics and Increased Current Amplitudes in hASIC1a-G212

HASIC1a-G212 and -D212 were expressed in CHO cells, and their function was assessed by whole-cell patch-clamp. The pH dependence of activation was measured by activating ASICs by a short exposure to acidic solutions of different pH once every 40 s from a conditioning pH of 7.4 that was applied between the acidic stimulations. Typical current traces are shown in Figure 2A. The pH dependence of activation of the hASIC1a current is shown in Figure 2B. The pH of half-maximal activation (pH_50_) was not different, with pH_50_ of hASIC1a-D212 of 6.47 ± 0.02 (*n* = 8) and pH_50_ of hASIC1a-G212 of 6.51 ± 0.04 (*n* = 6; *p* = 0.74). Similarly to the human clone, the mouse ASIC1a (mASIC1a) carries a Gly at the homologous position. The pH_50_ was not changed in mASIC1a by the G212D mutation (pH_50_ = 6.75 ± 0.02, *n* = 4) compared to the pH_50_ of mASIC1a WT (G212, 6.69 ± 0.02, *n* = 9, *p* = 0.46, Figure 2C). These experiments were carried out at an extracellular Ca^2+^ concentration of 2 mM. Lowering the extracellular Ca^2+^ concentration in the stimulation solution is known to shift the pH curve of ASIC1a to more alkaline values, possibly because there is a competition between Ca^2+^ and protons (Babini et al., 2002). We confirm here such a shift in the pH dependence of activation for both, ASIC1a-G212 and -D212, when the extracellular Ca^2+^ concentration was lowered to 100 nM (Figure 2D; *p* < 0.001, the dotted lines indicate the respective pH dependence in the presence of 2 mM extracellular Ca^2+^ from Figure 2B for comparison). The pH_50_ values were not different, with 6.80 ± 0.04 (hASIC1a-D212, *n* = 5) and 6.85 ± 0.02 (hASIC1a-G212, *n* = 6, *p* = 0.75). The simplest kinetic scheme that can describe ASIC function, contains three states, the closed, open and desensitized state, as illustrated in Figure 2E (Blanchard and Kellenberger, 2011). When closed ASICs are exposed to a moderately acidic solution, they can enter the non-conducting desensitized state without apparent opening. This transition is called SSD. Changes in the pH dependence of SSD can affect the fraction of channels that are activated by an acidic pH. We have determined the pH dependence of SSD by exposing ASIC-expressing cells for 1 min to different conditioning pH values in the range of pH7.8–6.8, each time followed by a short exposure to pH5.0 to activate the channels. The pH5.0-induced current amplitude is plotted in Figure 2F as a function of the conditioning pH. This experiment showed a small acidic shift of the SSD pH dependence if Asp212 is replaced by Gly. The midpoint of the SSD pH dependence (pHD_50_) was 7.17 ± 0.01 (hASIC1a-D212, *n* = 5) and 7.09 ± 0.02 (hASIC1a-G212, *n* = 5, *p* < 0.05). In mouse ASIC1a, these values were 7.49 ± 0.02 (mASIC1a-G212D, *n* = 5, Figure 2G) and 7.35 ± 0.01 (mASIC1a WT, *n* = 7, *p* < 0.0001), thus reproducing qualitatively the shift observed in hASIC1a.

We had observed that we needed to transfect cells with less cDNA coding for hASIC1a-G212 than with that coding for hASIC1a-D212 to obtain similar current amplitudes. When cells were transfected with the same amount of DNA, the current amplitudes were ~20-fold greater with G212 (Figure 2H, *p* < 0.05). To distinguish whether this difference was due to an effect on the expression of the channel or on its function, we compared the total and the cell surface expression of ASIC1a-G212 and -D212 from Western blots (Figures 2I,J). This showed stronger bands of hASIC1a-G212 for the total and the cell surface expression (Figure 2I), and the quantification indicated that the surface-expressed hASIC1a-D212 protein amounted to ~60% of the -G212 protein (Figure 2J, *p* < 0.01). hASIC1a-G212 has, therefore, a ~10-fold higher ratio of the current/number of channels at the cell surface than hASIC1a-D212. Since the residue 212 is located quite far from the pore, it is unlikely that it influences the unitary conductance. Indeed, unitary current amplitudes of hASIC1a-D212 (Alijevic and Kellenberger, 2012), and rat ASIC1a which carries a Gly residue at the corresponding position (Zhang and Canessa, 2002) are highly similar. The slower open-desensitized transition in hASIC1a-G212, suggested by the slower current decay (Figure 2A), underlies some of the increase of macroscopic peak current amplitudes. The replacement of Asp212 by Gly increases likely in addition the fraction of membrane-resident channels that open upon acidification.

**Figure 3:**
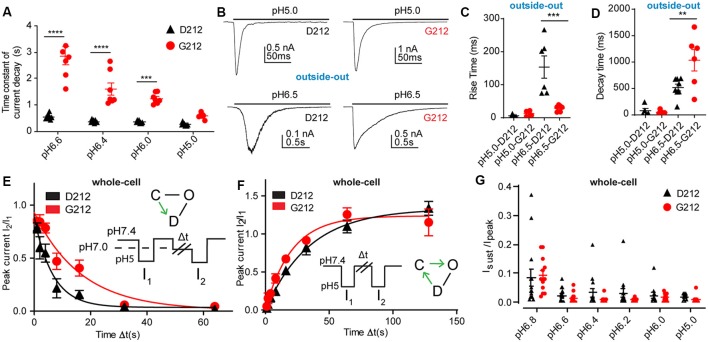
Slower current decay kinetics in hASIC1a-G212. All data in this figure are from hASIC1a expressed in CHO cells, black symbols represent hASIC1a-D212, red symbols hASIC1a-G212; **(A,E–G)**, measured with whole-cell patch-clamp; **(B–D)**, measured from excised, outside-out patches. **(A)** Time constant of current decay, obtained from single exponential fits at the pH conditions indicated, *n* = 7–8. ***p < 0.001; ****p < 0.0001, different between hASIC1a-D212 and -G212. **(B)** Representative traces showing ASIC1a currents from excised outside-out patches, induced by acidification to pH6.5 or pH5.0, by using an ultra-rapid perfusion system. **(C)** Rise time of current appearance (= time to pass from 10% to 90% of maximal peak amplitude), *n* = 5–6. **(D)** Current decay time, *n* = 5–8. **p < 0.01, ***p < 0.001, determined with ANOVA, followed by Tukey post-test. **(E)** The kinetics of desensitization at pH7.0, without apparent opening (thus likely the closed—desensitized transition), was determined with the protocol illustrated in the inset. The duration of the exposure to pH7.4 was 40 s, that to pH5.0 5 s, while exposure to pH7.0 of different durations Δt was used. The current ratio I_2_/I_1_ is plotted as a function of Δt. The solid lines represent single exponential fits, with τ = 8.0 ± 1.5 s (hASIC1a-D212, *n* = 11) and τ = 17.4 ± 3.5 s (hASIC1a-G212, *n* = 11, *p* > 0.05). **(F)** The kinetics of recovery from desensitization was determined by the protocol shown in the inset. Two subsequent steps to pH5.0 were separated by an exposure of varying duration (Δt) to pH7.4. After the second exposure, the cell was exposed for 40 s to pH7.4 before starting the next protocol. The current ratio I_2_/I_1_ is plotted as a function of Δt. The solid lines represent single exponential fits to the kinetics of hASIC1a-D212 (τ = 41.2 ± 5.2 s, *n* = 8) and hASIC1a-G212 (τ = 37.2 ± 10.4 s, *n* = 11, *p* < 0.05). **(G)** Sustained/peak current ratio of hASIC1a-D212 and -G212 at the indicated pH, *n* = 9–14. The sustained current was measured during the last second of the 10-s acidification. There was no significant difference between the I_sust_/I_peak_ ratio of hASIC1a-D212 and -G212 at any pH measured (Two-way ANOVA, followed by Sidak post-test).

As mentioned above, a slower time course of the current decay in G212 was obvious from the current traces (Figure 2A). Analysis of the time constant of current decay by fitting this part of the current trace to a single exponential shows a clear slowing of the desensitization kinetics in the pH range 6.6–6.0 when Asp212 was replaced by Gly (Figure 3A). At pH5.0, there was no significant difference in the current decay kinetics. To measure the current kinetics at high temporal resolution, a series of experiments were carried out with a piezo-driven solution change system on excised outside-out patches from CHO cells expressing ASIC1a-D212 or -G212 (Figures 3B–D). Upon stimulation with pH5.0, the kinetics of current appearance, measured as (10%–90%) rise time, were not different between the two hASIC1a variants, with 6.0 ± 1.8 ms (Figures 3B,C, hASIC1a-D212, *n* = 4) and 12.9 ± 3.3 ms (hASIC1a-G212, *n* = 6, *p* < 0.05). At the stimulation pH6.5, the current appearance was slower in hASIC1a-D212 (*p* < 0.001). The kinetics of current decay was rapid in both, hASIC1a-G212 and -D212 at pH5.0 (Figure 3D), with time constants of ~25 ms. Since the current decay had two exponential components in some patches, and one in others, we express the time course of current decay as decay time (time to pass from 90% to 10% of the current amplitude). At pH5.0, the kinetics were similarly fast for both ASIC types, while at pH6.5, the current decay was slower in G212 (*p* < 0.01). These observations suggest that in the pH range 6.6–6, the open-desensitized transition is slower in hASIC1a-G212 as compared to -D212. We wanted to know whether other transitions in and out of the desensitized state were also different between the two channels. To determine the kinetics of the closed-desensitized transition, channels were exposed for different durations to the conditioning pH 7.0, before activation of the non-desensitized channels by pH5.0 (Figure 3E). These experiments showed that the closed-desensitized kinetics are slower in hASIC1a-G212 as compared to -D212 (*p* < 0.05). The recovery from desensitization protocol (Figure 3F) showed, however, no difference between the two channel types (*p* > 0.05). ASIC1a current responses are mostly transient, and only a very small sustained current persists at the end of a 5-s acidification. If a sustained current appears, it is due to channels that can exit the desensitized state and return, either directly or *via* the closed state, to the open state. A difference in the sustained current/peak current (I_sust_/I_peak_) ratio would, therefore, indicate a difference in the rate leaving the desensitized state. The I_sust_/I_peak_ ratio, measured under different pH conditions, showed no significant difference between hASIC1a-D212 and -G212 (Figure 3G). Taken together, the kinetic analysis indicates a slower entry to the desensitized state from the open and the closed state for hASIC1a-G212. The acidic shift of the pH dependence of SSD suggests that the desensitized state is energetically slightly less favorable in hASIC1a-G212 compared to -D212.

**Figure 4:**
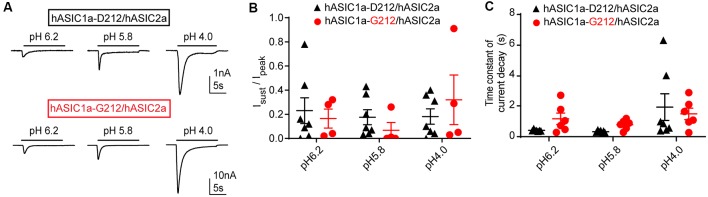
Properties of ASIC1a/ASIC2a heteromers. The data are from CHO cells transfected with hASIC1a-D212/hASIC2a (black symbols) and hASIC1a-G212/hASIC2a (red symbols), measured with whole-cell patch-clamp. **(A)** Current traces of hASIC1a-D212/hASIC2a (top) and hASIC1a-G212/hASIC2a activation (bottom), obtained with stimulation pH values as indicated, from conditioning pH 7.4. The acidic pH was applied for 10 s with a sweep interval of 40 s. **(B)** Sustained/peak current ratio at the indicated stimulation pH, *n* = 4–7. The sustained current was measured during the last second of the 10-s acidification. There was no significant difference between the I_sust_/I_peak_ ratio of the two channel types at any pH measured (Two-way ANOVA, followed by Sidak post-test). **(C)** Time constant of current decay, as obtained from single exponential fits, at the indicated pH, *n* = 6–7. There was no significant difference in the time constant of current decay between the two channel types at any pH measured (Two-way ANOVA, followed by Sidak post-test).

We have also tested whether the I_sust_/I_peak_ ratio and the desensitization kinetics are different between heteromeric hASIC1a-D212/hASIC2a and hASIC1a-G212/hASIC2a channels. Heteromeric channels were obtained by co-transfection of hASIC1a and hASIC2a constructs. IpH5.8/IpH4.0 current ratios of 0.43 ± 0.08 (hASIC1a-D212/hASIC2a, *n* = 7) and 0.41 ± 0.10 (hASIC1a-G212/hASIC2a, *n* = 5) indicated that mostly heteromeric channels were expressed (Joeres et al., 2016; Alijevic et al., 2018). Representative current traces are shown in Figure 4A. The I_sust_/I_peak_ ratio was not different between the two types of heteromers (Figure 4B). Although there was a tendency towards slower desensitization kinetics in hASIC1a-G212/hASIC2a as compared to hASIC1a-D212/hASIC2a at pH6.2 and 5.8, this difference was not statistically significant (Figure 4C). Likely, the influence of the residue at position ASIC1a-212 on current kinetics was reduced by the additional presence of ASIC2a subunits.

### Small Differences in the Pharmacology and Modulation

**Figure 5:**
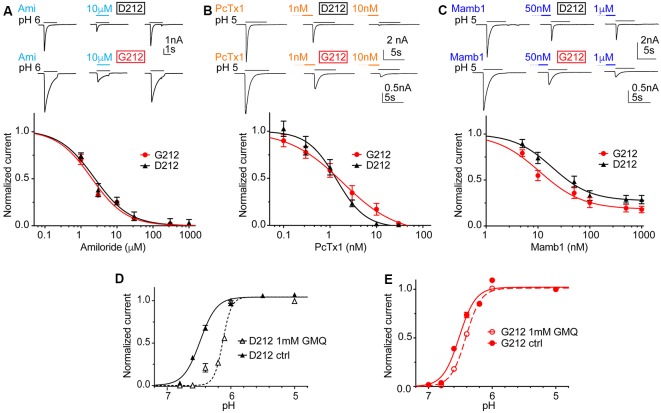
Unchanged pharmacology in hASIC1a-D212. All data in this figure are from hASIC1a expressed in CHO cells, measured with whole-cell patch-clamp; black symbols represent hASIC1a-D212, red symbols hASIC1a-G212. Conditioning pH was 7.4 in all experiments, and lines are fits to a Hill equation.** (A)** Top, representative current traces of ASIC1a activated by pH6.0 for 3 s without inhibitor, in the presence of 10 μM amiloride, and after washout of amiloride. Bottom, amiloride inhibition curve, plotting the current as a function of amiloride concentration, normalized to the response in the absence of amiloride, *n* = 4–5. **(B)** Top, representative current traces obtained under control conditions or after pre-administration of PcTx1 at the indicated concentrations. Bottom, PcTx1 inhibition curve, *n* = 6; PcTx1 was pre-applied in the conditioning pH solution during 60 s, before activating ASICs with pH5.0 during 5 s. **(C)** Top, representative current traces obtained under control conditions or after pre-administration of Mambalgin1 at the indicated concentrations. Bottom, Mambalgin1 inhibition curve, *n* = 7–8; Mambalgin1 was pre-applied in the conditioning pH solution during 60 s, before activating ASICs with pH5.0 during 5 s. **(D,E)** pH dependence of activation in the presence and absence of 1 mM 2-guanidine-4-methylquinazoline (GMQ) in the acidic solution; dotted lines and empty symbols represent the condition with GMQ. **(D)** D212, *n* = 10–11, *p* < 0.0001. **(E)** G212, *n* = 11–12, *p* < 0.01.

In order to investigate possible differences in pharmacology, we measured the IC_50_ of the general ENaC/DEG channel inhibitor amiloride, as well as IC_50_ values of the two ASIC toxin inhibitors Psalmotoxin1 (PcTx1) and Mambalgin1 (Mamb1). Amiloride and PcTx1 did not distinguish between hASIC1a-D212 and -G212, as illustrated by IC_50_ values of 1.91 ± 0.03 μM (hASIC1a-D212) and 1.54 ± 0.41 μM (hASIC1a-G212) for amiloride (Figure 5A, *n* = 4–5, *p* = 0.45) and 1.19 ± 0.26 nM (D212) and 1.83 ± 0.92 nM (G212, *n* = 5–6, *p* = 0.49) for PcTx1 (Figure 5B). Mambalgin1 had a 2-fold higher IC_50_ in D212, with 21.7 ± 4.3 nM (D212) and 10.5 ± 1.7 nM (G212, *n* = 7, *p* = 0.03; Figure 5C). GMQ can activate ASIC3 at physiological pH7.4 (Yu et al., 2010). We have previously shown that GMQ induces in ASIC1a an acidic shift of the pH dependence of activation, in contrast to its effects on ASIC3 (Alijevic and Kellenberger, 2012). Figures 5D,E show that 1 mM GMQ induces a significant lowering of the pH_50_ in both hASIC1a-D212 [6.47 ± 0.02 (ctrl) and 6.20 ± 0.05 (GMQ, *n* = 6–8, *p* < 0.0001)] and hASIC1a-G212 [6.51 ± 0.04 (ctrl) and 6.44 ± 0.02 (GMQ, *n* = 6–12, *p* < 0.01)]. The shift in pH dependence appears, however, to be substantially smaller in G212 as compared to D212. We have recently identified GMQ derivatives that also induced shifts in the pH dependence of ASIC channels (Alijevic et al., 2018). Two of these compounds, GMQ and 5-phenyl-2guanidinopyridine were tested on hASIC1a-D212 and -G212. These compounds affected the pH dependence and the maximal current amplitude in the same way (Supplementary Figure S2).

### Altered Dependence of Current Kinetics on Cl^−^ Concentration

**Figure 6:**
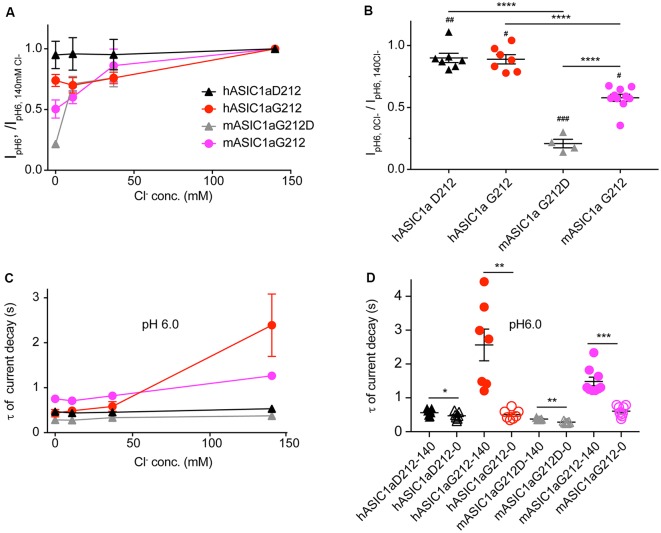
The chloride concentration affects the current decay kinetics in human and mouse ASIC1a-G212. All data in this figure are from human or mouse ASIC1a expressed in CHO cells, measured with whole-cell patch-clamp; black symbols represent hASIC1a-D212, red symbols hASIC1a-G212, gray symbols mASIC1a-G212D and purple symbols mASIC1a-G212. Conditioning pH was 7.4 in all experiments. Chloride in the extracellular solutions (conditioning and stimulating) was replaced in part or completely by SCN^−^, as indicated. **(A)** The I_pH6.0_ measured at a given Cl^−^ concentration is normalized to the I_pH6.0_ measured in normal extracellular medium containing 140 mM Cl^−^ in the same cell, shown for the four constructs, *n* = 4–5. **(B)** The ratio of the I_pH6.0_ measured in 140 mM SCN^−^/0 Cl^−^/I_pH6.0_ measured in 140 mM Cl^−^/0 SCN^−^ is plotted. Statistical analysis with paired *t*-tests showed that in all four constructs, the change in ion affected the current amplitude: ^#^*p* < 0.05, ^##^*p* < 0.01, ^###^*p* < 0.001. Differences between constructs (ANOVA followed by Tukey post-test) are indicated, **p < 0.01, ****p < 0.0001. **(C)** Time constants of current decay, derived from single-exponential fits, obtained with different anion concentrations as indicated, at pH6, *n* = 4–5. **(D)** Time constants of current decay, shown for each of the constructs at 0 and 140 mM Cl^−^, at pH6. Differences between the two ion conditions of a given construct are *p < 0.05, **p < 0.01 or ***p < 0.001, determined by ANOVA followed by Tukey post-test.

Since residue 212 is located in the proximity of the site where a Cl^−^ ion was found in the desensitized and open ASIC structures (Jasti et al., 2007; Gonzales et al., 2009; Baconguis and Gouaux, 2012; Baconguis et al., 2014), and mutations of nearby residues were shown to affect the ASIC1a current decay kinetics (Kusama et al., 2010), we measured ASIC1a currents in solutions in which the extracellular Cl^−^ was in part or completely replaced by SCN^−^. Figure 6A illustrates that the current amplitudes measured at pH6.0 decreased when the Cl^−^ concentration was reduced. Comparison of the two extreme conditions, by normalizing for each cell the I_pH6.0_ amplitude obtained at 0 mM Cl^−^/140 mM SCN^−^ to that obtained with 140 mM Cl^−^/0 mM SCN^−^, shows that the current amplitudes were lower in the Cl^−^-free condition, and that this current decrease was stronger in the mouse as compared to the human channels (Figure 6B). The reasons for this change in amplitude may involve changes in current kinetics and pH dependence. The current decay kinetics at pH6.0 were similarly rapid at 0, 11 and 37 mM Cl^−^ in all four ASIC1a constructs, and were slowed down for hASIC1a-G212 at 140 mM Cl^−^ (Figures 6C,D). In all these four channel variants, the current decay kinetics were different between the two extreme conditions (0 mM Cl^−^/140 mM SCN^−^ compared to 140 mM Cl^−^/0 mM SCN^−^, Figure 6D). The ratio of the time constants between the two ionic conditions (τ_140 mM Cl_^−^/ τ_140 mM SCN_^−^) was however small in mouse and hASIC1a carrying an Asp at position 212 and was significantly increased in hASIC1a-G212 (*p* < 0.01).

### Confirmation in the hASIC1a-G212 Background of Results of Previous Mutagenesis Studies

**Figure 7:**
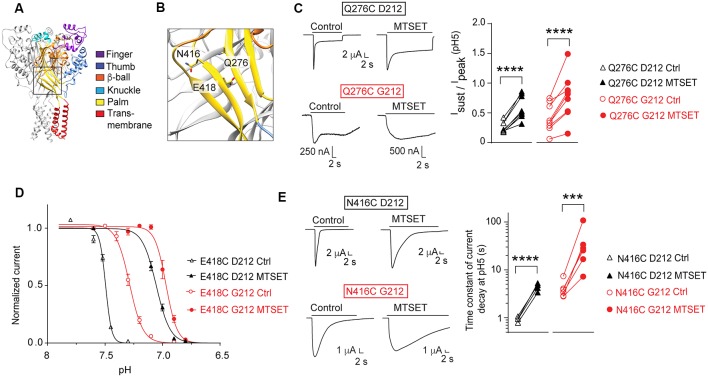
Conserved effects of mutations in the palm. All experiments of this figure are from hASIC1a expressed in *Xenopus* oocytes, voltage-clamped to −60 mV. **(A,B)** Overview and detailed image of a structural model of hASIC1a, based on the crystal structure of cASIC1a (Baconguis et al., 2014). The domains of one subunit are indicated by different colors, as in Figure 1A. **(C)** Left, representative current traces of the mutant Q276C in the background of hASIC1a-D212 (top) or -G212 (bottom), as indicated, before and after MTSET exposure (1 mM, 3 min). ASIC currents were induced by changing from the conditioning pH7.4 to the stimulation pH5.0. Right, the I_sust_/I_peak_ ratio at pH5.0 (*n* = 8–10) is shown. For Q276C D212C, the I_sust_/I_peak_ ratio was measured during the last 2 s of the acid perfusion; the duration of the pH5.0 perfusion was the same in the control and MTSET condition. For Q276C G212, this ratio was measured at 16–18 s after the start of the pH5.0 perfusion. **(D)** SSD pH dependence of the mutant E418C before (open symbols) or after MTSET exposure (filled symbols, 1 mM 3 min, *n* = 4–7). Normalized current amplitudes are plotted as function of the conditioning pH. Two-way ANOVA showed that MTSET induced an acidic shift of the pH dependence in both backgrounds (*p* < 0.0001). **(E)** Left, representative current traces of the mutant N416C in the background of hASIC1a-D212 (top) or -G212 (bottom), before and after MTSET exposure (1 mM, 3 min). ASIC currents were induced by changing from the conditioning pH7.4 to the stimulation pH5.0. Right, the time constant of the current decay measured at pH 5.0, is shown before and after exposure to MTSET (*n* = 5–6). For **(C,E)**, ***p < 0.001; ****p < 0.0001; paired *t*-test.

Our laboratory has carried out several studies in the background of hASIC1a-D212 that analyzed the functional consequences of mutations in a number of channel domains. We have constructed here some key mutants in the background of hASIC1a-G212 and tested whether the effect of these mutations was conserved in the hASIC1a-G212 background. In a study addressing the conformational changes in the palm (Roy et al., 2013), we had investigated a large number of palm mutants. In these experiments we had shown among other observations that exposure to MTSET induced in Q276C an increase of the I_sust_/I_peak_ ratio, in E418C an acidic shift of the pH dependence of SSD (see also Liechti et al., 2010), and in N416C a slowing of the kinetics of current decay. Figures 7A,B show the localization of these residues in the ASIC1a protein. When these three mutations were generated in hASIC1a-G212, the time course of current decay was as expected slower than in the original mutants made in hASIC1a-D212, as illustrated by the representative traces in Figures 7C,E. Exposure to MTSET induced however qualitatively the same effects as those observed in the original study, thus an increase of the I_sust_/I_peak_ ratio of Q276C (Figure 7C), an acidic shift of the SSD pH dependence curve in E418C (Figure 7D), and a slowing of the current decay kinetics of N416C (Figure 7E).

**Figure 8:**
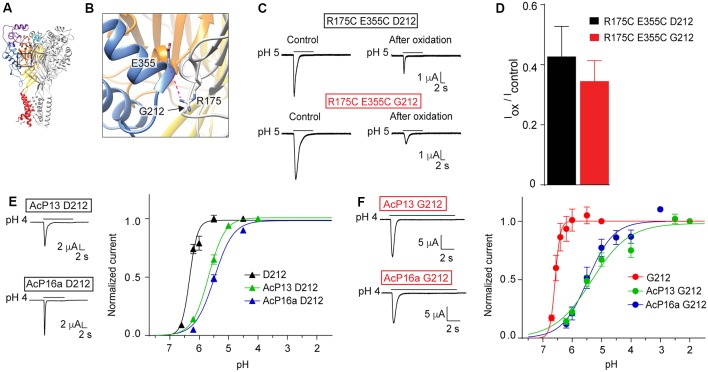
Mutations in and close to the acidic pocket. All experiments of this figure are from hASIC1a expressed in *Xenopus* oocytes, voltage-clamped to −60 mV. **(A,B)** Overview and detailed view of a structural model of hASIC1a, based on the crystal structure of cASIC1a (Baconguis et al., 2014), showing the subunit interface between the thumb of one subunit (blue) and the palm domain of an adjacent subunit (gray). The predicted hydrogen bond Arg175 – Glu355 is shown as a pink dashed line. **(C)** Representative current traces of R175C E355C D212 and R175C E355C G212 mutants before and after the oxidation treatment with copper phenanthroline. The oxidation treatment was done exactly as described (Gwiazda et al., 2015). **(D)**, The I_pH5.0 (after oxidation)_/I_pH5.0 (before oxidation)_ ratio is shown (*n* = 5–9; **E**). Left, representative current traces of mutants AcP13 D212 and AcP16a D212, measured in response to an acidification to pH 4.0 from a conditioning pH of 8.0 and 7.6, respectively. Right, pH dependence of activation of hASIC1a-D212, and the two mutants AcP13 and AcP16a in the hASIC1a-D212 background, (*n* = 2–12). **(F)** Same as in **(E)**, however in the G212 background, *n* = 5–6. **(E,F)** The mutants AcP13 and AcP16a contain the following mutations: AcP13: E97Q/E177Q/D183N/D237N/E238Q/D303N/E315Q/E321Q/H329N/E344Q/D347N/D351N/E355Q; AcP16a: E97Q/E113Q/E177Q/D183N/E235Q/D237N/E238Q/D303N/E315Q/E321Q/H329N/E340Q/E344Q/D347N/D351N/E355Q. The conditioning pH was 7.4, except for AcP13, where it was 8.0, and AcP16a (pH 7.6).

In a study investigating ASIC1a intersubunit interactions, we had shown that among others, disulfide bond formation between E355C of one subunit and R175C of a neighboring subunit, forces the channel in a non-conducting state (Gwiazda et al., 2015). Glu355 and Arg175 are located in close proximity to residue 212 (Figures 8A,B). A key observation in this study was the current decrease after exposure to copper phenanthroline that promotes the formation of disulfide bonds. Here we show that the inhibition by copper phenanthroline is very similar in the R175C/E355C mutant made in the Gly212 background (Figures 8C,D).

In another study, we had shown that simultaneous neutralization of a high number of acidic residues in the acidic pocket induced an acidic shift of the pH dependence of activation, but still allowed the activation of transient currents by extracellular acidification. We had concluded that the protonation events in the acidic pocket are not essential for ASIC activation, but have rather a modulatory role (Vullo et al., 2017). Here we have constructed two of these mutants, termed AcP13 (containing the mutations E97Q/E177Q/D183N/D237N/E238Q/D303N/E315Q/E321Q/H329N/E344Q/D347N/D351N/E355Q) and AcP16a (containing the mutations E97Q/E113Q/E177Q/D183N/E235Q/D237N/E238Q/D303N/E315Q/E321Q/H329N/E340Q/E344Q/D347N/D351N/E355Q) in the hASIC1a-G212 background. We show that these mutations in the Gly212 background produced current kinetics similar to those of the corresponding WT (Figures 8E,F), as had been observed in the original study. These mutations induced in both backgrounds an acidic shift of the pH dependence of activation (Figures 8E,F). These control experiments confirm that the effects of mutations investigated in these studies did not depend on the presence of a Gly or Asp residue at position 212.

In a very recent study, we have shown evidence for binding of the peptide Phe-Arg-Arg-Phe-amide, an ASIC modulator, to the palm of ASIC1a (Bargeton et al., 2019). This peptide generates a small sustained current in ASIC1a and induces an acidic shift of the pH dependence of SSD. The study was done in the background of D212. We have shown in the same study that this peptide induced a smaller shift of the pH dependence of SSD in hASIC1a-G212 as compared to hASIC1a-D212, but that the peptide-induced sustained current level was indistinguishable between hASIC1a-G212 and hASIC1a-D212 (Bargeton et al., 2019).

### Conservation of Voltage-Clamp Fluorometry Findings in the hASIC1a-G212 Background

**Figure 9:**
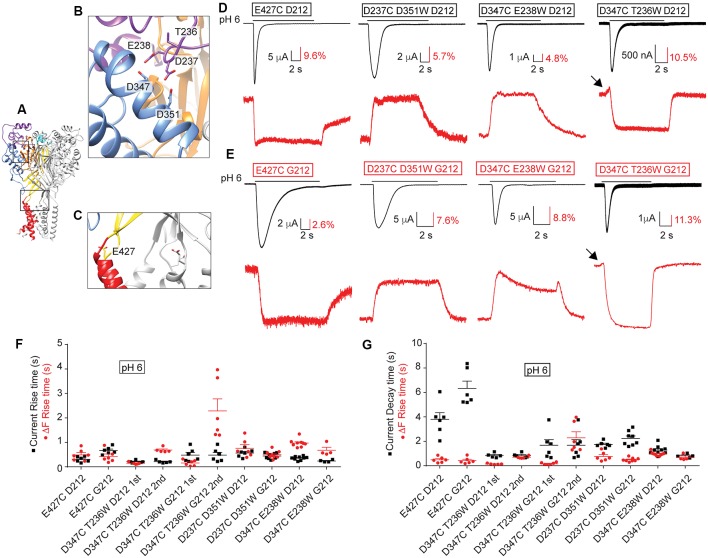
Conserved ΔF signal patterns in voltage-clamp fluorometry (VCF) experiments. All experiments of this figure are from hASIC1a expressed in *Xenopus* oocytes, voltage-clamped to −40 mV. **(A–C)** Different views of the structural model of hASIC1a described in legend of Figure 7, showing in **(B)** a close-up of the acidic pocket, in **(C)** a close-up of the wrist, indicating the positions of the residues that were mutated to Cys to attach the fluorophore. **(D)** Representative current (black) and fluorescence (red) traces of Cys and Cys-Trp mutants in the hASIC1a-D212 background. **(E)** Traces of the corresponding Cys and Cys-Trp mutants in the background of hASIC1a-G212. **(D,E)** Fluorophore-labeled oocytes were exposed to the stimulation pH6.0 from a conditioning pH7.4. Black arrows point to fast ΔF components. **(F,G)** Scatter dot plots comparing the fluorescence rise time (red) with the current rise time **(F)** or decay time **(G)** in response to an acidic stimulation at pH6.0 from a conditioning pH7.4, *n* = 4–9.

We have previously used VCF to describe conformational changes in ASIC1a (Bonifacio et al., 2014; Vullo et al., 2017). In these studies, the fluorophore was attached to engineered Cys residues that had been placed in several different locations within the ASIC1a ectodomain (Figures 9A–C), and the ionic current and the fluorescence signal were simultaneously recorded, as illustrated in Figures 9D,E. One position, E427C in hASIC1a (Figure 9C), had previously been employed in mASIC1a (mASIC1a-E425C; Passero et al., 2009). The shape of the VCF signal with the fluorophore anchored at this position was highly similar in mASIC1a and in the hASIC1a-D212 (Passero et al., 2009; Bonifacio et al., 2014) and hASIC1a-G212 background (Figures 9D,E). We have determined the kinetics of the currents and fluorescence changes (ΔF) as rise or decay times (= time to pass from 10% to 90% of the total amplitude, or inverse). The current rise time (black symbols) and ΔF rise time (red) are plotted in Figure 9F, indicating for the E427C mutant in both backgrounds rapid current and ΔF kinetics. In the G212 background, the kinetics of current appearance and decay of E427C were slower than in the D212 background [*p* < 0.01 and *p* < 0.001, respectively (One-Way ANOVA followed by Tukey post-test)]. The ΔF rise time was considerably faster than the current decay of E427C in both backgrounds (Figure 9G). To determine whether the relation between current and ΔF kinetics in a given mutant is different between the D212 and G212 backgrounds, we have determined in each experiment the ratio of the current rise time/ΔF rise time, and of the current decay time/ΔF rise time. The comparison of these values showed that the relation between the current and ΔF kinetics was not different between E427C D212 and E427C G212 (One-Way ANOVA, followed by Tukey post-test, *p* > 0.05).

In our recent VCF study, we had combined the placement of a fluorophore *via* the introduction of a Cys residue with the insertion of a quenching group by a mutation to Trp nearby in the channel (Vullo et al., 2017). In this study, we had observed ΔF with one or two components. We have now generated three of these mutations in the background of hASIC1a-G212. We show here that for these mutants the ΔF pattern in both hASIC1a backgrounds is highly similar (Figures 9D,E). The quantitative analysis of the kinetics of these mutants is shown in Figures 9F,G. A statistical comparison of the current/ΔF kinetic ratios, carried out in the same way as described above for the E427C mutant, showed no significant effect of the residue at position 212 on the relation between current and ΔF kinetics in any of these mutants.

## Discussion

Several laboratories working with hASIC1a have used for many years, and are still using, a variant containing an Asp residue at the place of Gly212 as their WT clone. We found here the following differences in biophysical properties between hASIC1a-D212 and the WT hASIC1a-G212: (1) the midpoint of SSD is shifted by 0.08 pH units to more acidic values in hASIC1a-G212; (2) with the same amount of transfected DNA, hASIC1a-G212 shows ~20-fold higher current amplitudes, and a ~2-fold higher cell-surface expression than -D212; (3) the current decay kinetics are slower in hASIC1a-G212 (~5-fold at pH close to the pH_50_, and ~2-fold at more acidic pH in whole-cell experiments). These kinetics depend on the extracellular Cl^−^ concentration in both channel types; the Cl^−^ dependence is however considerably stronger in hASIC1a-G212; and (4) the kinetics of the closed → desensitized transition is 2-fold slower in hASIC1a-G212 as compared to -D212. Based on our observations, it is obvious that the hASIC1a-D212 clone needs to be replaced in future studies by the hASIC1a WT. Other parameters, such as the sustained current fraction and the kinetics of recovery from desensitization are not different between hASIC1a-G212 and -D212.

The higher current amplitude per amount of transfected DNA with hASIC1a-G212 should not have caused differences in the results in recombinant expression studies, since in such studies, the quantity of DNA is generally adjusted in order to obtain current amplitudes that are big enough to be detected, and small enough to allow a reliable measurement. The faster kinetics of current decay in hASIC1a-D212 lead to a shorter window of activity than would be observed with -G212, and the small alkaline shift in the pH dependence of SSD can, under certain pH conditions, decrease the number of channels available for opening. The faster closed-desensitized transition in hASIC1a-D212 would add to this effect. Due to these differences, the functional impact of ASIC1a was somewhat underestimated in cellular studies with hASIC1a-D212.

One aim of the present work was to test whether results obtained with studies using hASIC1a-D212 as “WT” remain valid. With regard to modulation of ASIC1a function by compounds, we show here that the three tested inhibitors decrease currents of hASIC1a-D212 and-G212 in a concentration-dependent manner, with indistinguishable IC_50_ values between the two channel types in the case of amiloride and PcTx1, and with a 2-fold decreased IC_50_ value for hASIC1a-G212 in the case of Mambalgin1. GMQ leads to an acidic shift of the ASIC1a pH dependence (Alijevic and Kellenberger, 2012). This shift is observed in both ASIC1a types, it appears, however, to be smaller in hASIC1a-G212 as compared to -D212. An acidic shift of the activation pH dependence by GMQ was recently shown in rat ASIC1a, which contains a Gly at position 212 (Besson et al., 2017), further demonstrating that the existence of this shift does not depend on Asp212. The experiments with these different compounds show that qualitatively, the effects are the same in hASIC1a-G212 and -D212. Based on this analysis, we conclude that results obtained in pharmacological studies with most other compounds, or studies with modulators on hASIC1a-D212, are likely also valid if the binding site of the compound under investigation is not close to 212. There may however exist some differences in absolute IC_50_ values, as shown for Mambalgin1.

Many structure-function studies have been carried out with hASIC1a-D212. Since the pH dependence is only marginally different between hASIC1a-G212 and -D212, and the transient nature of H^+^-induced currents is conserved in both hASIC1a types, we think that the presence of Asp212 should not much change the effects of other mutations. To test this interpretation, we have repeated key experiments of structure-function studies done in our laboratory in the past with hASIC1a-D212, this time with the mutants made in the Gly212 background. As expected, these mutants showed slower current decay kinetics when constructed in the hASIC1a-G212 background. The effects of the mutations observed in the hASIC1a-D212 background were however conserved in the -G212 background, indicating strongly that the conclusions of our original studies remain valid. In several studies, we had employed VCF to analyze conformational changes during channel activity (Bonifacio et al., 2014; Gwiazda et al., 2015; Vullo et al., 2017). We have now constructed four mutants used in these studies in the hASIC1a-G212 background, and have repeated the VCF experiments. We show that the pattern of the fluorescence signal and the relation between current and ΔF kinetics are conserved, indicating that the conclusions made in the previous studies remain valid.

Given that the most striking effect of exchanging Asp and Gly at position 212 is the change in the kinetics of current decay, we reasoned that this substitution should affect the transition from the open to the desensitized state. Gly212 is located in the β-ball, at a subunit interface in the proximity of the lower ends of the thumb α helices α4 and α5. Comparison of the crystal structures of cASIC1a in the closed, open and desensitized states indicates a substantial rearrangement of this interface in the transition from the closed to the open, but not from the open to the desensitized state (Gonzales et al., 2009; Baconguis et al., 2014; Yoder et al., 2018). Therefore, there is no obvious structural explanation for the difference in the current decay kinetics between hASIC1a-G212 and -D212.

A Cl^−^ binding site in close proximity of residue 212 was found in the open and desensitized but not in the closed ASIC1a structure (Jasti et al., 2007; Gonzales et al., 2009; Baconguis and Gouaux, 2012; Baconguis et al., 2014; Yoder and Gouaux, 2018; Yoder et al., 2018). It was shown that the chloride concentration affects the ASIC current decay kinetics (Kusama et al., 2010, 2013), and that mutations of the predicted Cl^−^ binding site disrupted the modulation of the current decay kinetics if introduced in ASIC1a (Kusama et al., 2010), and had a partial, or no effect if introduced into ASIC2a or ASIC3, respectively (Kusama et al., 2013). This indicates therefore that Cl^−^ likely binds to the proximity of position 212 to affect the current decay kinetics. We show here that the replacement of Cl^−^ by SCN^−^ accelerates the current decay kinetics in both, hASIC1a-D212 and -G212, but that this effect is significantly enhanced in hASIC1a-G212. Thus, the exchange of Gly and Asp at position 212 may not affect the binding of Cl^−^ itself, but rather its consequences on current decay kinetics. This is in line with the conclusion of the previous study, that both, SCN^−^ and Cl^−^ bind to the same binding site in ASIC1a, and that SCN^−^ even binds with higher affinity than Cl^−^, but cannot induce a modulatory effect and therefore acts as a kind of competitive antagonist (Kusama et al., 2010).

In conclusion, we have compared the function of hASIC1a-D212, a mutant that has in many studies been used as the hASIC1a WT, with the WT hASIC1a-G212, and show that these two channels are similar in many functional aspects, including the biophysical properties, the pharmacology and the effect of mutations, but that hASIC1a-D212 has faster current decay kinetics than hASIC1a-G212.

## Contribution to the Field Statement

We have recently realized that the acid-sensing channel 1a (ASIC1a) construct that has been used as WT in studies by many laboratories is indeed a rare mutant. In the present study we have compared the functional and pharmacological properties of the mutant and the WT channel. We show that the mutant channel differs in its current kinetics and current expression from the WT. The effects of the tested pharmacological agents were however highly similar in both channel types. In the past, many structure-function studies have been carried out based on this mutant construct. We have therefore generated key mutations of previous studies in the WT background, and have tested their effects. Our analysis shows that the effects of these mutations are conserved. Our study validates therefore the conclusions of the previous studies. Since an important part of the current understanding of the structure-function relationship and of the pharmacology of ASIC1a is based on studies using this mutant as “WT”, the validation of these studies is critical. Our results are therefore important for the field of ASIC and Epithelial Na^+^ channel pharmacology and function, and for the structure-function relationship of these channels.

## Data Availability

All datasets generated for this study are included in the manuscript and/or the Supplementary Files.

## Ethics Statement

This study was carried out in accordance with the recommendations of Swiss federal law on animal welfare, controlled by the veterinary service of the canton de Vaud. The protocol was approved by the veterinary service of the canton de Vaud.

## Author Contributions

All authors designed together the project. AV, SV and ZP carried out the experiments. AV, SV, ZP, OA and SK wrote the manuscript.

## Conflict of Interest Statement

The authors declare that the research was conducted in the absence of any commercial or financial relationships that could be construed as a potential conflict of interest.

## Footnotes

^1^exac.broadinstitute.org

## Abbreviations

ASIC: Acid-Sensing Ion Channel
cASIC1: chicken ASIC
cDNA: complementary deoxyribonucleic acid
CHO: Chinese hamster ovary
hASIC1a: human ASIC1a
GMQ: 2-Guanidine-4-methylquinazoline
Mamb1: Mambalgin1
mASIC1a: mouse ASIC1a
MD: Molecular dynamics
pH_50_: pH of half-maximal activation
pHD_50_: pH of half-maximal desensitization
PcTx1: Psalmotoxin1
SSD: Steady-state desensitization
VCF: voltage-clamp fluorometry
WT: wild-type.
