# Supplementary material for: Accelerated Current Decay Kinetics of a Rare Human Acid-Sensing ion Channel 1a Variant That Is Used in Many Studies as Wild Type
Source: Front Mol Neurosci. 2019 May 24;12:133. doi: 10.3389/fnmol.2019.00133 (PMC6542941; doi:10.3389/fnmol.2019.00133)
Supplement: Supplementary file 1 [file Data_Sheet_1.PDF]

Table S1. List of articles using recombinant hASIC1a, with indication of the residue at position 212

In the column "Category", the notations of the different letters are as follows. "A", Identified as hASIC1a-D212 based on one of the following indications, 1) Indication "Obtained from D.P. Corey", 2) GenBank accession number U78181, 3) Reference as the source of the clone to an article that uses the hASIC1a clone obtained from D.P. Corey; "B", Identified based on the GenBank accession number NM_001095 which describes hASIC1a having a Gly at position 212; "C", Origin of the isoform of hASIC1a, either D212 or G212 is not clear from the indications in the article; "D", Cloned from human brain; "E", Human brain tissue sample (B, D, E have a Gly at position 212). In some of the cited studies, only a part of the data was obtained from human ASIC1a. The attributions in this table were done to the best of our knowledge. We apologize in case articles are wrongly attributed to a category.

Title of the article	Category	hASIC1a-D212	hASIC1a-G212	Reference
BNaC1 and BNaC2 constitute a new family of human neuronal sodium channels related to degenerins and epithelial sodium channels	A	×		(Garcia-Anoveros et al., 1997)
pH Alterations "Reset" Ca^{2+} Sensitivity of Brain Na^{+} Channel 2, a Degenerin/Epithelial Na^{+} Ion Channel, in Planar Lipid Bilayers	A	×		(Berdiev et al., 2001)
Up-regulation of Acid-gated Na^{+} Channels (ASICs) by Cystic Fibrosis Transmembrane Conductance Regulator Co-expression in Xenopus Oocytes	A	×		(Ji et al., 2002)
Acid-sensing Ion Channels in Malignant Gliomas	A	×		(Berdiev et al., 2003)
cAMP-dependent protein kinase phosphorylation of the acid-sensing ion channel-1 regulates its binding to the protein interacting with C-kinase-1	C			(Leonard et al., 2003)

Overexpression of acid-sensing ion channel 1a in transgenic mice increases acquired fear-related behaviour	C			(Wemmie et al., 2004)
Subunit-Dependent High-Affinity Zinc Inhibition of Acid-Sensing Ion Channels	C			(Chu et al., 2004)
Selective Regulation of Acid-sensing Ion Channel 1 by Serine Proteases	A	×		(Poirot et al., 2004)
Characterization of Acid-sensing Ion Channels in Dorsal Horn Neurons of Rat Spinal Cord	C			(Wu et al., 2004)
Coupling between NMDA Receptor and Acid-Sensing Ion Channel Contributes to Ischemic Neuronal Death	C			(Gao et al., 2005)
Calcium-permeable Acid-sensing Ion Channel Is a Molecular Target of the Neurotoxic Metal Ion Lead	C			(Wang et al., 2006)
A Gating Mutation in the Internal Pore of ASIC1a	A	×		(Pfister et al., 2006)
Nafamostat mesilate reversibly blocks acid-sensing ion channel currents	D		×	(Ugawa et al., 2007)
Potentiation of acid-sensing ion channels by sulfhydryl compounds	C			(Cho and Askwith, 2007)
Direct visualization of the trimeric structure of the ASIC1a channel, using AFM imaging	A	×		(Carnally et al., 2008)
Presynaptic Release Probability Is Increased in Hippocampal Neurons From ASIC1 Knockout Mice	C			(Cho and Askwith, 2008)

Endogenous Arginine-Phenylalanine-Amide-related Peptides Alter Steady-state Desensitization of ASIC1a	C			(Sherwood and Askwith, 2008)
Restoring Acid-Sensing Ion Channel-1a in the Amygdala of Knock-Out Mice Rescues Fear Memory But Not Unconditioned Fear Responses	C			(Coryell et al., 2008)
Inherent Dynamics of the Acid-Sensing Ion Channel 1 Correlates with the Gating Mechanism	A	×		(Yang et al., 2009)
Native and recombinant ASIC1a receptors conduct negligible Ca^{2+} entry	B		×	(Samways et al., 2009)
Identification of Protein Domains That Control Proton and Calcium Sensitivity of ASIC1a	C			(Sherwood et al., 2009)
ASIC2 Subunits Target Acid-Sensing Ion Channels to the Synapse via an Association with PSD-95	C			(Zha et al., 2009)
PICK1 regulates the trafficking of ASIC1a and acidotoxicity in a BAR domain lipid binding-dependent manner	A	×		(Jin et al., 2010)
Acid-sensing ion channel (ASIC) 1a undergoes a height transition in response to acidification	A	×		(Yokokawa et al., 2010)
Demonstration of a Direct Interaction between σ-1 Receptors and Acid-Sensing Ion Channels	A	×		(Carnally et al., 2010)

A Combined Computational and Functional Approach Identifies New Residues Involved in pH-dependent Gating of ASIC1a	A	×		(Liechti et al., 2010)
Acid-sensing ion channels in acidosis-induced injury of human brain neurons	E		×	(Li et al., 2010)
The Contact Region between Three Domains of the Extracellular Loop of ASIC1a Is Critical for Channel Function	A	×		(Bargeton and Kellenberger, 2010)
Extracellular Spermine Exacerbates Ischemic Neuronal Injury through Sensitization of ASIC1a Channels to Extracellular Acidosis	C			(Duan et al., 2011)
N-Glycosylation of Acid-Sensing Ion Channel 1a Regulates Its Trafficking and Acidosis-Induced Spine Remodelling	C			(Jing et al., 2012)
Highly Conserved Salt Bridge Stabilizes Rigid Signal Patch at Extracellular Loop Critical for Surface Expression of Acid-sensing Ion Channels	C			(Yang et al., 2012)
Black mamba venom peptides target acid-sensing ion channels to abolish pain	B		×	(Diochot et al., 2012)
Subtype-specific Modulation of Acid-sensing Ion Channel (ASIC) Function by 2-Guanidine-4-methylquinazoline	A	×		(Alijevic and Kellenberger, 2012)
PI3-kinase/Akt Pathway-Regulated Membrane Insertion of Acid-Sensing Ion Channel 1a Underlies BDNF-Induced Pain Hypersensitivity	C			(Duan et al., 2012)

Structure of the Acid-sensing ion channel 1 in complex with the gating modifier Psalmotoxin 1	C			(Dawson et al., 2012)
Molecular Mechanism of Constitutive Endocytosis of Acid-Sensing Ion Channel 1a and Its Protective Function in Acidosis-Induced Neuronal Death	C			(Zeng et al., 2013)
Conformational Changes in the Lower Palm Domain of ASIC1a Contribute to Desensitization and RFamide Modulation	E		×	(Frey et al., 2013)
Protonation controls ASIC1a activity via coordinated movements in multiple domains	A	×		(Bonifacio et al., 2014)
Role of ASIC1 in the development of chronic hypoxia-induced pulmonary hypertension	C			(Nitta et al., 2014)
Fluorescence resonance energy transfer analysis of subunit assembly of the ASIC channel	B		×	(Bartoi et al., 2014)
Identification of a Novel Protein Complex Containing ASIC1a and GABAA Receptors and Their Interregulation	C			(Zhao et al., 2014)
Chloroquine impairs visual transduction via modulation of acid sensing ion channel 1a	C			(Li et al., 2014b)
A Method for Activation of Endogenous Acid-sensing Ion Channel 1a (ASIC1a) in the Nervous System with High Spatial and Temporal Precision	C			(Li et al., 2014a)

Inhibition of acid-sensing ion channel currents by propofol in rat dorsal root ganglion neurons	C			(Lei et al., 2014)
Ion conduction and selectivity in acid-sensing ion channel 1	B		×	(Yang and Palmer, 2014)
Site-Specific Fluorescence Spectrum Detection and Characterization of hASIC1a Channels upon Toxin Mambalgin-1 binding in Live Mammalian Cells	C			(Wen et al., 2015)
Activation of acid-sensing ion channels by localized proton transient reveals their role in proton signalling	A	×		(Zeng et al., 2015)
The Human Acid-Sensing Ion Channel ASIC1a: Evidence for a Homotetrameric Assembly State at the Cell Surface	A	×		(van Bemmelen et al., 2015)
Extracellular Subunit Interactions Control Transitions between Functional States of Acid-sensing Ion Channel 1a	A	×		(Gwiazda et al., 2015)
Atomic force microscopy imaging reveals the formation of ASIC/ENaC cross-clade ion channels	A	×		(Jeggle et al., 2015)
Tissue acidosis induces neuronal necroptosis via ASIC1a channel independent of its ionic conduction	B		×	(Wang et al., 2015)
NS383 Selectively Inhibits Acid-Sensing Ion Channels Containing 1a and 3 Subunits to Reverse Inflammatory and Neuropathic Hyperalgesia in Rats	C			(Munro et al., 2016)

Deactivation kinetics of acid-sensing ion channel 1a are strongly pH-sensitive	A	×	(MacLean and Jayaraman, 2017)
Proton and non-proton activation of ASIC channels	A	×	(Gautschi et al., 2017)
Conformational dynamics and role of the acidic pocket in ASIC pH-dependent gating	A	×	(Vullo et al., 2017)
Potent neuroprotection after stroke afforded by a double-knot spider-venom peptide that inhibits acid-sensing ion channel 1a	C		(Chassagnon et al., 2017)
Bile acids potentiate proton-activated currents in Xenopus laevis oocytes expressing human acid-sensing ion channel (ASIC1a)	A	×	(Ilyaskin et al., 2017)
Human ASIC1a mediates stronger acid-induced responses as compared with mouse ASIC1a	C		(Xu et al., 2018)
Heteroarylguanidines as Allosteric Modulators of ASIC1a and ASIC3 Channels	A	×	(Alijevic et al., 2018)
Cryo-EM structure of the ASIC1a – mambalgin-1 complex reveals that the peptide toxin mambalgin-1 inhibits acid-sensing ion channels through an unusual allosteric effect	C		(Sun et al., 2018)

Supplementary references

Alijevic, O., Hammoud, H., Vaithia, A., Trendafilov, V., Bollenbach, M., Schmitt, M., et al. (2018). Heteroarylguanidines as Allosteric Modulators of ASIC1a and ASIC3 Channels. *ACS Chem Neurosci* 9(6)**,** 1357-1365. doi:10.1021/acschemneuro.7b00529.

Alijevic, O., and Kellenberger, S. (2012). Subtype-specific modulation of acid-sensing ion channel (ASIC) function by 2-guanidine-4-methylquinazoline. *J Biol Chem* 287(43)**,** 36059-36070. doi: 10.1074/jbc.M112.360487.

Bargeton, B., and Kellenberger, S. (2010). The contact region between three domains of the extracellular loop of ASIC1a is critical for channel function. *J Biol Chem* 285(18)**,** 13816-13826. doi: M109.086843 [pii]10.1074/jbc.M109.086843.

Bartoi, T., Augustinowski, K., Polleichtner, G., Grunder, S., and Ulbrich, M.H. (2014). Acid-sensing ion channel (ASIC) 1a/2a heteromers have a flexible 2:1/1:2 stoichiometry. *Proc Natl Acad Sci U S A* 111(22)**,** 8281-8286. doi: 10.1073/pnas.1324060111.

Berdiev, B.K., Mapstone, T.B., Markert, J.M., Gillespie, G.Y., Lockhart, J., Fuller, C.M., et al. (2001). pH alterations "reset" Ca2+ sensitivity of brain Na+ channel 2, a degenerin/epithelial Na+ ion channel, in planar lipid bilayers. *J Biol Chem* 276(42)**,** 38755-38761. doi: 10.1074/jbc.M107266200.

Berdiev, B.K., Xia, J., McLean, L.A., Markert, J.M., Gillespie, G.Y., Mapstone, T.B., et al. (2003). Acid-sensing ion channels in malignant gliomas. *J Biol Chem* 278(17)**,** 15023-15034. doi: 10.1074/jbc.M300991200.

Bonifacio, G., Lelli, C.I., and Kellenberger, S. (2014). Protonation controls ASIC1a activity via coordinated movements in multiple domains. *J Gen Physiol* 143(1)**,** 105-118. doi: 10.1085/jgp.201311053.

Carnally, S.M., Dev, H.S., Stewart, A.P., Barrera, N.P., Van Bemmelen, M.X., Schild, L., et al. (2008). Direct visualization of the trimeric structure of the ASIC1a channel, using AFM imaging. *Biochem Biophys Res Commun* 372(4)**,** 752-755. doi: S0006-291X(08)01031-0 [pii]10.1016/j.bbrc.2008.05.100.

Carnally, S.M., Johannessen, M., Henderson, R.M., Jackson, M.B., and Edwardson, J.M. (2010). Demonstration of a direct interaction between sigma-1 receptors and acid-sensing ion channels. *Biophys J* 98(7)**,** 1182-1191. doi: 10.1016/j.bpj.2009.12.4293.

Chassagnon, I.R., McCarthy, C.A., Chin, Y.K., Pineda, S.S., Keramidas, A., Mobli, M., et al. (2017). Potent neuroprotection after stroke afforded by a double-knot spider-venom peptide that inhibits acid-sensing ion channel 1a. *Proc Natl Acad Sci U S A* 114(14)**,** 3750-3755. doi: 10.1073/pnas.1614728114.

Cho, J.H., and Askwith, C.C. (2007). Potentiation of acid-sensing ion channels by sulfhydryl compounds. *Am J Physiol Cell Physiol* 292(6)**,** C2161-2174. doi: 10.1152/ajpcell.00598.2006.

Cho, J.H., and Askwith, C.C. (2008). Presynaptic release probability is increased in hippocampal neurons from ASIC1 knockout mice. *J Neurophysiol* 99(2)**,** 426-441. doi: 10.1152/jn.00940.2007.

Chu, X.P., Wemmie, J.A., Wang, W.Z., Zhu, X.M., Saugstad, J.A., Price, M.P., et al. (2004). Subunit-dependent high-affinity zinc inhibition of acid-sensing ion channels. *J Neurosci* 24(40)**,** 8678-8689.

Coryell, M.W., Wunsch, A.M., Haenfler, J.M., Allen, J.E., McBride, J.L., Davidson, B.L., et al. (2008). Restoring Acid-Sensing Ion Channel-1a in the Amygdala of Knock-Out Mice Rescues Fear Memory But Not Unconditioned Fear Responses. *Journal of Neuroscience* 28(51)**,** 13738-13741. doi: 10.1523/JNEUROSCI.3907-08.2008.

Dawson, R.J., Benz, J., Stohler, P., Tetaz, T., Joseph, C., Huber, S., et al. (2012). Structure of the Acid-sensing ion channel 1 in complex with the gating modifier Psalmotoxin 1. *Nat Commun* 3**,** 936. doi: 10.1038/ncomms1917.

Diochot, S., Baron, A., Salinas, M., Douguet, D., Scarzello, S., Dabert-Gay, A.S., et al. (2012). Black mamba venom peptides target acid-sensing ion channels to abolish pain. *Nature* 490(7421)**,** 552-555. doi: 10.1038/nature11494.

Duan, B., Liu, D.S., Huang, Y., Zeng, W.Z., Wang, X., Yu, H., et al. (2012). PI3-kinase/Akt pathway-regulated membrane insertion of acid-sensing ion channel 1a underlies BDNF-induced pain hypersensitivity. *J Neurosci* 32(18)**,** 6351-6363. doi: 10.1523/JNEUROSCI.4479-11.2012.

Duan, B., Wang, Y.Z., Yang, T., Chu, X.P., Yu, Y., Huang, Y., et al. (2011). Extracellular spermine exacerbates ischemic neuronal injury through sensitization of ASIC1a channels to extracellular acidosis. *J Neurosci* 31(6)**,** 2101-2112. doi: 10.1523/JNEUROSCI.4351-10.2011.

Frey, E.N., Pavlovicz, R.E., Wegman, C.J., Li, C., and Askwith, C.C. (2013). Conformational changes in the lower palm domain of ASIC1a contribute to desensitization and RFamide modulation. *PLoS One* 8(8)**,** e71733. doi: 10.1371/journal.pone.0071733.

Gao, J., Duan, B., Wang, D.G., Deng, X.H., Zhang, G.Y., Xu, L., et al. (2005). Coupling between NMDA receptor and acid-sensing ion channel contributes to ischemic neuronal death. *Neuron* 48(4)**,** 635-646. doi: 10.1016/j.neuron.2005.10.011.

Garcia-Anoveros, J., Derfler, B., Nevillegolden, J., Hyman, B.T., and Corey, D.P. (1997). BNaC1 and BNaC2 constitute at new family of human neuronal sodium channels related to degenerins and epithelial sodium channels. *Proc. Natl. Acad. Sci. USA* 94(4)**,** 1459-1464.

Gautschi, I., van Bemmelen, M.X., and Schild, L. (2017). Proton and non-proton activation of ASIC channels. *PLoS One* 12(4)**,** e0175293. doi: 10.1371/journal.pone.0175293.

Gwiazda, K., Bonifacio, G., Vullo, S., and Kellenberger, S. (2015). Extracellular Subunit Interactions Control Transitions between Functional States of Acid-sensing Ion Channel 1a. *J Biol Chem* 290(29)**,** 17956-17966. doi: 10.1074/jbc.M115.641688.

Ilyaskin, A.V., Diakov, A., Korbmacher, C., and Haerteis, S. (2017). Bile acids potentiate proton-activated currents in Xenopus laevis oocytes expressing human acid-sensing ion channel (ASIC1a). *Physiol Rep* 5(3). doi: 10.14814/phy2.13132.

Jeggle, P., Smith, E.S., Stewart, A.P., Haerteis, S., Korbmacher, C., and Edwardson, J.M. (2015). Atomic force microscopy imaging reveals the formation of ASIC/ENaC cross-clade ion channels. *Biochem Biophys Res Commun* 464(1)**,** 38-44. doi: 10.1016/j.bbrc.2015.05.091.

Ji, H.L., Jovov, B., Fu, J., Bishop, L.R., Mebane, H.C., Fuller, C.M., et al. (2002). Up-regulation of acid-gated Na(+) channels (ASICs) by cystic fibrosis transmembrane conductance regulator co-expression in Xenopus oocytes. *J Biol Chem* 277(10)**,** 8395-8405. doi: 10.1074/jbc.M109465200.

Jin, W., Shen, C., Jing, L., Zha, X.M., and Xia, J. (2010). PICK1 regulates the trafficking of ASIC1a and acidotoxicity in a BAR domain lipid binding-dependent manner. *Mol Brain* 3**,** 39. doi: 10.1186/1756-6606-3-39.

Jing, L., Chu, X.P., Jiang, Y.Q., Collier, D.M., Wang, B., Jiang, Q., et al. (2012). N-glycosylation of acid-sensing ion channel 1a regulates its trafficking and acidosis-induced spine remodeling. *J Neurosci* 32(12)**,** 4080-4091. doi: 10.1523/JNEUROSCI.5021-11.2012.

Lei, Z., Li, X., Wang, G., Fei, J., Meng, T., Zhang, X., et al. (2014). Inhibition of acid-sensing ion channel currents by propofol in rat dorsal root ganglion neurons. *Clin Exp Pharmacol Physiol* 41(4)**,** 295-300. doi: 10.1111/1440-1681.12215.

Leonard, A.S., Yermolaieva, O., Hruska-Hageman, A., Askwith, C.C., Price, M.P., Wemmie, J.A., et al. (2003). cAMP-dependent protein kinase phosphorylation of the acid-sensing ion channel-1 regulates its binding to the protein interacting with C-kinase-1. *Proc Natl Acad Sci U S A* 100(4)**,** 2029-2034. doi: 10.1073/pnas.252782799.

Li, M., Inoue, K., Branigan, D., Kratzer, E., Hansen, J.C., Chen, J.W., et al. (2010). Acid-sensing ion channels in acidosis-induced injury of human brain neurons. *J Cereb Blood Flow Metab* 30(6)**,** 1247-1260. doi: 10.1038/jcbfm.2010.30.

Li, T., Yang, Y., and Canessa, C.M. (2014a). A method for activation of endogenous acid-sensing ion channel 1a (ASIC1a) in the nervous system with high spatial and temporal precision. *J Biol Chem* 289(22)**,** 15441-15448. doi: 10.1074/jbc.M114.550012.

Li, X., Fei, J., Lei, Z., Liu, K., Wu, J., Meng, T., et al. (2014b). Chloroquine impairs visual transduction via modulation of acid sensing ion channel 1a. *Toxicol Lett* 228(3)**,** 200-206. doi: 10.1016/j.toxlet.2014.05.008.

Liechti, L.A., Berneche, S., Bargeton, B., Iwaszkiewicz, J., Roy, S., Michielin, O., et al. (2010). A combined computational and functional approach identifies new residues involved in pH-dependent gating of ASIC1a. *J Biol Chem* 285(21)**,** 16315-16329. doi: M109.092015 [pii]10.1074/jbc.M109.092015.

MacLean, D.M., and Jayaraman, V. (2017). Deactivation kinetics of acid-sensing ion channel 1a are strongly pH-sensitive. *Proc Natl Acad Sci U S A* 114(12)**,** E2504-E2513. doi: 10.1073/pnas.1620508114.

Munro, G., Christensen, J.K., Erichsen, H.K., Dyhring, T., Demnitz, J., Dam, E., et al. (2016). NS383 Selectively Inhibits Acid-Sensing Ion Channels Containing 1a and 3 Subunits to Reverse Inflammatory and Neuropathic Hyperalgesia in Rats. *CNS Neurosci Ther* 22(2)**,** 135-145. doi: 10.1111/cns.12487.

Nitta, C.H., Osmond, D.A., Herbert, L.M., Beasley, B.F., Resta, T.C., Walker, B.R., et al. (2014). Role of ASIC1 in the development of chronic hypoxia-induced pulmonary hypertension. *Am J Physiol Heart Circ Physiol* 306(1)**,** H41-52. doi: 10.1152/ajpheart.00269.2013.

Pfister, Y., Gautschi, I., Takeda, A.N., van Bemmelen, M., Kellenberger, S., and Schild, L. (2006). A gating mutation in the internal pore of ASIC1a. *J Biol Chem* 281(17)**,** 11787-11791. doi: 10.1074/jbc.M513692200.

Poirot, O., Vukicevic, M., Boesch, A., and Kellenberger, S. (2004). Selective regulation of acid-sensing ion channel 1 by serine proteases. *J Biol Chem* 279(37)**,** 38448-38457. doi: 10.1074/jbc.M407381200.

Samways, D.S., Harkins, A.B., and Egan, T.M. (2009). Native and recombinant ASIC1a receptors conduct negligible Ca^{2+} entry. *Cell Calcium* 45(4)**,** 319-325. doi: 10.1016/j.ceca.2008.12.002.

Sherwood, T., Franke, R., Conneely, S., Joyner, J., Arumugan, P., and Askwith, C. (2009). Identification of protein domains that control proton and calcium sensitivity of ASIC1a. *J Biol Chem* 284(41)**,** 27899-27907. doi: 10.1074/jbc.M109.029009.

Sherwood, T.W., and Askwith, C.C. (2008). Endogenous arginine-phenylalanine-amide-related peptides alter steady-state desensitization of ASIC1a. *J Biol Chem* 283(4)**,** 1818-1830. doi: M705118200 [pii]10.1074/jbc.M705118200.

Sun, D., Yu, Y., Xue, X., Pan, M., Wen, M., Li, S., et al. (2018). Cryo-EM structure of the ASIC1a-mambalgin-1 complex reveals that the peptide toxin mambalgin-1 inhibits acid-sensing ion channels through an unusual allosteric effect. *Cell Discov* 4**,** 27. doi: 10.1038/s41421-018-0026-1.

Ugawa, S., Ishida, Y., Ueda, T., Inoue, K., Nagao, M., and Shimada, S. (2007). Nafamostat mesilate reversibly blocks acid-sensing ion channel currents. *Biochem Biophys Res Commun* 363(1)**,** 203-208. doi: 10.1016/j.bbrc.2007.08.133.

van Bemmelen, M.X., Huser, D., Gautschi, I., and Schild, L. (2015). The Human Acid-Sensing Ion Channel ASIC1a: Evidence for a Homotetrameric Assembly State at the Cell Surface. *PLoS One* 10(8)**,** e0135191. doi: 10.1371/journal.pone.0135191.

Vullo, S., Bonifacio, G., Roy, S., Johner, N., Berneche, S., and Kellenberger, S. (2017). Conformational dynamics and role of the acidic pocket in ASIC pH-dependent gating. *Proc Natl Acad Sci U S A* 114(14)**,** 3768-3773. doi: 10.1073/pnas.1620560114.

Wang, W., Duan, B., Xu, H., Xu, L., and Xu, T.L. (2006). Calcium-permeable acid-sensing ion channel is a molecular target of the neurotoxic metal ion lead. *J Biol Chem* 281(5)**,** 2497-2505. doi: 10.1074/jbc.M507123200.

Wang, Y.Z., Wang, J.J., Huang, Y., Liu, F., Zeng, W.Z., Li, Y., et al. (2015). Tissue acidosis induces neuronal necroptosis via ASIC1a channel independent of its ionic conduction. *Elife* 4. doi: 10.7554/eLife.05682.

Wemmie, J.A., Coryell, M.W., Askwith, C.C., Lamani, E., Leonard, A.S., Sigmund, C.D., et al. (2004). Overexpression of acid-sensing ion channel 1a in transgenic mice increases acquired fear-related behavior. *Proc Natl Acad Sci U S A* 101(10)**,** 3621-3626. doi: 10.1073/pnas.0308753101.

Wen, M., Guo, X., Sun, P., Xiao, L., Li, J., Xiong, Y., et al. (2015). Site-specific fluorescence spectrum detection and characterization of hASIC1a channels upon toxin mambalgin-1 binding in live mammalian cells. *Chem Commun (Camb)* 51(38)**,** 8153-8156. doi: 10.1039/c5cc01418b.

Wu, L.J., Duan, B., Mei, Y.D., Gao, J., Chen, J.G., Zhuo, M., et al. (2004). Characterization of acid-sensing ion channels in dorsal horn neurons of rat spinal cord. *J Biol Chem* 279(42)**,** 43716-43724. doi: 10.1074/jbc.M403557200.

Xu, Y., Jiang, Y.Q., Li, C., He, M., Rusyniak, W.G., Annamdevula, N., et al. (2018). Human ASIC1a mediates stronger acid-induced responses as compared with mouse ASIC1a. *FASEB J* 32(7)**,** 3832-3843. doi: 10.1096/fj.201701367R.

Yang, H., Yu, Y., Li, W.G., Yu, F., Cao, H., Xu, T.L., et al. (2009). Inherent dynamics of the acid-sensing ion channel 1 correlates with the gating mechanism. *PLoS Biol* 7(7)**,** e1000151. doi: 10.1371/journal.pbio.1000151.

Yang, L., and Palmer, L.G. (2014). Ion conduction and selectivity in acid-sensing ion channel 1. *J Gen Physiol* 144(3)**,** 245-255. doi: 10.1085/jgp.201411220.

Yang, Y., Yu, Y., Cheng, J., Liu, Y., Liu, D.S., Wang, J., et al. (2012). Highly conserved salt bridge stabilizes rigid signal patch at extracellular loop critical for surface expression of acid-sensing ion channels. *J Biol Chem* 287(18)**,** 14443-14455. doi: 10.1074/jbc.M111.334250.

Yokokawa, M., Carnally, S.M., Henderson, R.M., Takeyasu, K., and Edwardson, J.M. (2010). Acid-sensing ion channel (ASIC) 1a undergoes a height transition in response to acidification. *FEBS Lett* 584(14)**,** 3107-3110. doi: 10.1016/j.febslet.2010.05.050.

Zeng, W.Z., Liu, D.S., Duan, B., Song, X.L., Wang, X., Wei, D., et al. (2013). Molecular mechanism of constitutive endocytosis of Acid-sensing ion channel 1a and its protective function in acidosis-induced neuronal death. *J Neurosci* 33(16)**,** 7066-7078. doi: 10.1523/JNEUROSCI.5206-12.2013.

Zeng, W.Z., Liu, D.S., Liu, L., She, L., Wu, L.J., and Xu, T.L. (2015). Activation of acid-sensing ion channels by localized proton transient reveals their role in proton signaling. *Scientific Reports* 5. doi: ARTN 14125 10.1038/srep14125.

Zha, X.M., Costa, V., Harding, A.M., Reznikov, L., Benson, C.J., and Welsh, M.J. (2009). ASIC2 subunits target acid-sensing ion channels to the synapse via an association with PSD-95. *J Neurosci* 29(26)**,** 8438-8446. doi: 10.1523/JNEUROSCI.1284-09.2009.

Zhao, D., Ning, N., Lei, Z., Sun, H., Wei, C., Chen, D., et al. (2014). Identification of a novel protein complex containing ASIC1a and GABAA receptors and their interregulation. *PLoS One* 9(6)**,** e99735. doi: 10.1371/journal.pone.0099735.

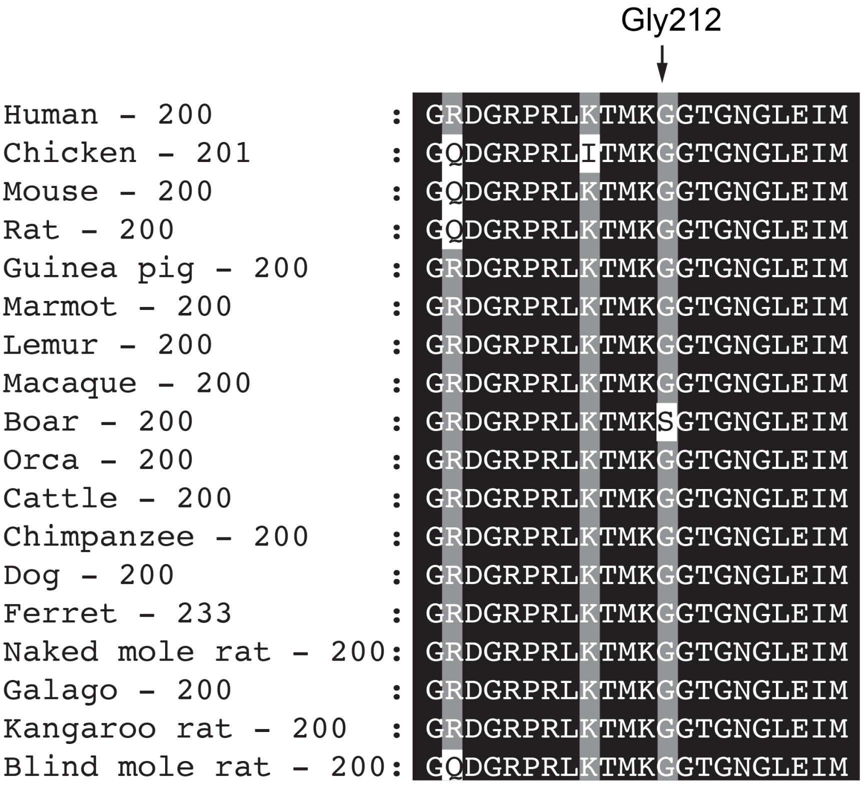

Figure S1. Conservation of Gly212 in ASIC1a. Alignment of ASIC1a from different species, as indicated. The number of the first displayed amino acid residue is indicated after the species name. Gly212 is highlighted in the alignment.

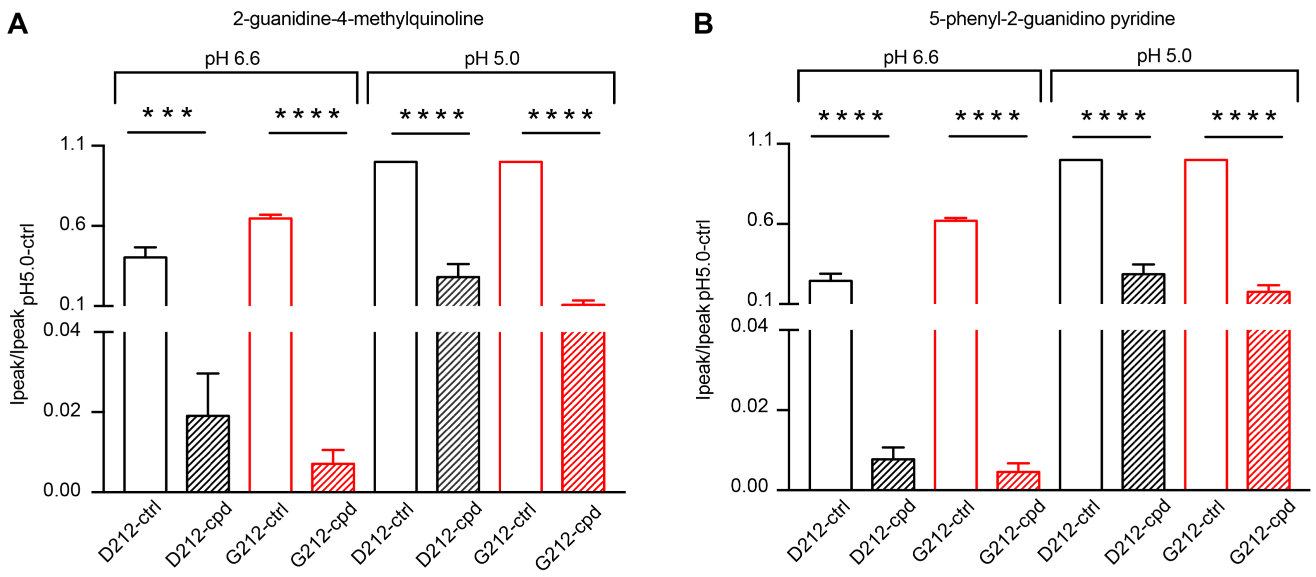

Figure S2. Effects of 2-guanidine-4-methylquinazoline derivatives are conserved between hASIC1a-D212 and –G212. Data are from whole-cell patch-clamp of CHO cells expressing these ASICs. Two compounds, 2-guanidine-4-methylquinoline **(A)** and 5-phenyl-2-guanidinopyridine **(B)** were tested at two pH conditions, pH6.6, where their effect is due to changes in the pH dependence and possibly in part to pore block, and at pH5.0, where their effect is mostly due to pore block. The two compounds were administered with the acidic solution for 5s at a concentration of 1 mM; ctrl, control; cpd, in presence of the compound. Current amplitudes were normalized to the one obtained with pH5.0 in the absence of compound in the same cell, n=5-8. The statistical significance of the drug effects was analyzed by two-way ANOVA followed by Sidak's post-test; ***, $p<0.001$, ****, $p<0.0001$.
